# The Association between Widowhood and Cognitive Function among Chinese Elderly People: Do Gender and Widowhood Duration Make a Difference?

**DOI:** 10.3390/healthcare9080991

**Published:** 2021-08-04

**Authors:** Nan Xiang, Erpeng Liu, Huwei Li, Xigang Qin, Hang Liang, Zhang Yue

**Affiliations:** 1 School of Public Administration, Zhongnan University of Economics and Law, Wuhan 430073, China; xiangnan512@outlook.com (N.X.); lihuwei_zuel@163.com (H.L.); 18426418748@163.com (X.Q.); lianghang304@163.com (H.L.); 2 Institute of Income Distribution and Public Finance, Zhongnan University of Economics and Law, Wuhan 430073, China; whubest@163.com

**Keywords:** widowhood, cognitive function, gender, widowhood duration, Chinese elderly people

## Abstract

Few studies have examined the effects of widowhood on cognitive function in Chinese elderly individuals. We conducted a longitudinal study to assess the association between widowhood and cognitive function and further explored gender differences in this association and the impact of widowhood duration. The analytical sample consisted of 5872 Chinese elderly people who participated in the Chinese Longitudinal Healthy Longevity Survey (CLHLS) and were followed up from 2005 to 2014. We used the Chinese version of the Mini-Mental State Examination (MMSE) to assess cognitive function. Widowhood duration was calculated from the self-reported year at which the spouse passed away. Multilevel growth models were employed to estimate the association between widowhood and cognitive function while adjusting for many demographic and socioeconomic characteristics. Widowhood status was associated with cognitive decline among Chinese elderly individuals after adjusting for covariates (B = −0.440, 95% CI −0.727 to −0.152), and this association was only statistically significant among men (B = −0.722, 95% CI −1.104 to −0.339). Being widowed for 5 years or less (B = −0.606, 95% CI −1.112 to −0.100), 16–20 years (B = −0.937, 95% CI −1.685 to −0.190), and 21+ years (B = −1.401, 95% CI −1.967 to −0.834) predicted worse cognitive function in men, while being widowed for more than 21+ years (B = −0.655, 95% CI −1.186 to −0.124) was associated with cognitive decline in women. More attention should be directed towards widowed men and long-term widowed elderly individuals.

## 1. Introduction

The trend of population ageing is developing particularly rapidly in China, which raises great concern for public health. The data from the seventh national census bulletin show that by 2020, there were 190.64 million Chinese elderly people over the age of 65, accounting for 13.50% of the total population [1]. It is predicted that by 2050, the number of Chinese elderly people will reach a peak of 488 million, representing 35.6% of the total population [2]. Because of the life expectancy gap between men and women, numerous elderly people will experience widowhood in the coming decades [3]. Widowed elderly individuals in China are predicted to reach 118.4 million by 2050, which is 2.5 times larger than the number in 2010 [4]. China is experiencing a period of accelerated aging, together with a large number of elderly people in unhealthy conditions. Taking the ageing population and the increasing life expectation together, China has become the fastest growing country in the world for dementia [5]. The number of individuals with dementia in China is estimated to be 9–10 million patients aged 65 years and older, and more than 60% of patients with dementia have Alzheimer’s disease [6]. The large number of elderly individuals suffering from cognitive decline will pose a heavy burden on medical costs without sufficient attention and policy interventions. Therefore, it is crucial to identify risk and protective factors for cognitive function.

The association between widowhood and cognitive function has been noticed in previous studies. However, the results have not been consistent. Several studies have confirmed that widowhood accelerates the decline in cognitive function in elderly individuals [7,8,9,10]. The stress model and marital resource model are often used to explain the mechanism between widowed and cognitive health. The stress model has indicated that stress-induced glucocorticoid secretion fueled by emotional trauma leads to hippocampal atrophy [11,12]. The marital resource model has suggested that marriage will help to obtain economic resources, social interaction, and social support, all of which are related to better cognitive function in old age [13,14]. Additionally, the interaction in the marriage relationship can provide elderly individuals with daily cognitive stimulation, which is of great importance to protect cognitive function [10]. Previous studies demonstrated that the widowed elderly without children or a supportive family might be in a worse health condition [15]. A few studies have suggested that widowhood is not related to cognitive function [16,17]. The effect of widowhood may vary with social and cultural background [18], and the salience of different mechanisms linking widowhood to health may depend on gender and local norms [19]. Therefore, gender differences in this relationship across countries are of great concern. Several studies have demonstrated that widowed men, but not women, had a worse health condition than married people [20,21]. However, widowhood has had a more serious negative impact on women of Mexican origin because they were more likely to adhere to traditional family roles [22], as well as in India with relatively conservative marriage culture and strict gender norms [23].

In addition, existing studies have suggested that the relationship between widowhood and health outcomes may be more complex than a simple binary effect (widowed versus non-widowed). Evidence in the Indian population showed that women widowed for 0–4 years and more than 10 years were more likely to experience depression, poor self-rated health, hypertension, and other health risk [23]. A longitudinal study from 2002 to 2014 suggested that the increased odds ratio of loneliness slightly decreased with the length of widowhood, but it was still significant, even after 40 years of bereavement [24]. Another study based on 17 years of follow-up data from 6766 elderly Americans found that there was a linear relationship between widowhood duration and decline in cognitive function [8]. Furthermore, a longitudinal research following 8 years in Korea found that widowed elderly individuals with widowhood duration of 4 to 6 years had a significantly steeper decline in cognitive function than nonwidowed elderly individuals [25]. Thus, it is necessary to take widowhood duration into consideration when examining the effect of widowhood on cognitive function.

Nevertheless, in contrast to many studies evaluating the widowhood effect in developed countries, little attention has been given to the relationship between widowhood and cognitive function in Chinese elderly individuals. Most available studies in China have been based on regional, cross-sectional data [26,27,28,29], with the exception of one study with only two years of follow-up [30]. A study in Taiwan demonstrated that widowed elderly individuals had higher odds of dementia than married individuals [26]. Similar results were reported in Beijing, Chengdu, Shanghai, and Xi’an [27]. However, an investigation in three Chinese communities showed that the significant association of being widowed with cognitive scores disappeared after adjustment for covariables [28]. In terms of gender differences, researchers found that widowhood increased the incidence of cognitive impairment in men, while the relationship was not statistically significant in women [29]. However, another study found that the effect of widowhood on memory decline was similar for men and women [30].

The findings of a widowhood effect on cognitive function and its gender differences have been contradictory across studies, and the importance of widowhood duration has not received sufficient attention in China [3]. Therefore, the present study attempted to address these gaps in several ways. In general, the main purpose of this study is to assess the association between widowhood and cognitive function and further explore gender differences in this association and the impact of widowhood duration. First, unlike most previous studies based on regional and cross-sectional data, we aimed to assess the widowhood effect on cognitive function through a nationwide population-based study with long-term follow-up data. Second, under the context of gender norms in China, gender differences were further analyzed in the present research. Third, we took widowhood duration into consideration, which has not received much attention in the literature. Through the above analysis, we want to raise concerns for the enormous population of widowed elderly in China and put forward corresponding policy suggestions for health intervention to improve their cognitive function.

## 2. Methods

### 2.1. Data Source and Study Population

Data were derived from the Chinese Longitudinal Healthy Longevity Survey (CLHLS), which was approved by the Research Ethics Committees of Duke University and Peking University. The CLHLS was first conducted in 1998, followed by six waves of surveys in 2000, 2002, 2005, 2008, 2011, and 2014. The population of the surveyed region accounts for 85.3% of the total population of the country, which can be regarded as a nationally representative sample. Participants in CLHLS were recruited by a targeted random sampling process, by which investigators first recruited an eligible centenarian interviewee in sampled city/county, and then matched a nonagenarian, an octogenarian, and three elders aged 65–79 nearby in the same street, village, or town [31]. The participants were surveyed through face-to-face interviews on a voluntary basis.

The first two CLHLS waves incorporated individuals aged 80 and above, while those aged 64–79 were enrolled beginning in 2002. Because of that, we excluded 1998 and 2000 waves because there was a difference in the age range of participants. We also found that the scale used to measure cognitive function in 2002 was slightly inconsistent with those used after 2005. To ensure the accuracy of the cognitive function measurement, the 2002 wave of data was also excluded. The present study sample included the 2005–2014 (2005, 2008, 2011, 2014) longitudinal datasets. A total of 7004 respondents who were aged 65 to 105 and participated in at least two waves were selected for analysis. Considering that we focused on widowhood status, we limited our sample to those who were married only once in order to minimize the impact of divorce or remarriage on the outcome variables of interest. As a result, 663 respondents who had divorced, remarried, separated, or never been married were excluded. A total of 469 respondents with significant missing data were not included in the present study. The final analytic sample consisted of 16,950 observations from 5872 respondents who completed, on average, approximately three waves of the survey (mean = 3.16, SD = 0.88). In total, 2695 respondents participated in two waves, accounting for 45.90%; 1148 respondents participated in three waves, accounting for 19.55%; and 2029 respondents participated in four waves, accounting for 34.55%.

### 2.2. Measures

#### 2.2.1. Cognitive Function

In the CLHLS, cognitive function was measured with the Chinese version of the Mini-Mental State Examination (MMSE) [32], which has been proven to be reliable and effective with Chinese elderly individuals [33,34]. It contains 24 items assessing orientation, attention, calculation, recall, and language, with total scores ranging from 0 to 30 (Supplementary Table S1). Higher scores indicate better cognitive function.

#### 2.2.2. Marital Status and Widowhood Duration

At the baseline survey and each follow-up wave of interviews, the respondents were asked to report their current marital status. As mentioned above, respondents included in the present study were either married or widowed. We therefore divided marital status into two categories: currently married elderly (reference group) and widowed elderly. If a respondent was widowed, the investigators further asked for the month/year of the spouse’s death. Widowhood duration was calculated from the self-reported year at which the spouse passed away.

#### 2.2.3. Covariates

Based on previous studies on cognitive function in elderly populations [35,36], the following four categories of risk factors were considered in the model as covariates: (1) demographic characteristics, including gender, age, education, and place of residence. (2) health-related factors, including ADL (activities of daily living), and chronic disease. The Katz scale was adopted to evaluate ADL in elderly individuals [37], which included bathing, dressing, bathroom use, indoor transferring, continence, and eating aspects of function. If any one of these activities could not be independently completed, the individuals were classified as limited in ADL. The presence of chronic disease was assessed for six diseases: cancer, hypertension, diabetes, heart attack, stroke, pneumonia, and tuberculosis. A score of 1 was recorded if the respondent reported having one or more of the six diseases, and otherwise, a score of 0 was recorded. (3) Financial condition, the respondents were asked “whether income is sufficient for paying daily expenses” (1 = yes; 0 = no) and “whether children (including all grandchildren and their spouses who live with you or not) provide cash (or in-kind) support” (1 = yes; 0 = no). (4) Lifestyle and living arrangements, lifestyle included whether they had smoked/drank/exercised in the past (1 = yes; 0 = no), and living arrangements were divided into living with others and living alone.

### 2.3. Statistical Analysis

We first present descriptive statistics in each wave, including means and standard deviations for continuous variables and number and proportion for categorical variables. Second, ANOVA tests were performed to compare the cognitive scores between different marital status groups. Third, considering that the four waves of longitudinal data might include time correlations within individuals, multilevel growth models were employed with maximum likelihood estimation (MLE), which is suitable for analyzing longitudinal data with repeated observations from the same individual over time and with panel attrition [38]. Two levels of panel data were used including the repeated measurement of cognitive function in level 1 nested across individuals in level 2. Marital status and other covariates that change with time were considered level 1 variables, including age, place of residence, ADL, chronic disease, income, children’s financial support, and living arrangements. Unchanged covariates, such as gender; education; or smoking, drinking, and exercising in the past were included in level 2. The age variable was centered to make the intercept meaningful. Model 1 reports the coefficient of widowhood status on cognitive function after adjusting for covariates. Model 2 takes widowhood duration as a continuous variable, while widowhood duration was further categorized into six groups (currently married as refence group) in Model 3. All analyses were conducted using the statistical software Stata15 (Stata Corp, College Station, TX, USA).

## 3. Results

Table 1 shows the general characteristics of the participants from 2005 to 2014. The average cognitive score of participants was 25.12 in 2005 and 24.25 in 2014. The widowed elderly accounted for 55.64% of the participants in 2005 and 56.34% of the participants in 2014. The average age of participant was 80.64 in 2005 and 83.33 in 2014. Women accounted for approximately 55% in each wave. Most of the participants had low education level, no ADL limitations, sufficient income, and financial support from children. There was an obvious decreased trend of participants living in rural areas. Additionally, the number of the elderly with chronic disease increased from 2005 to 2008. Most of the participates lived with others and exercised and drank but did not smoke in the past.

The cognitive function of the elderly individuals according to their marital status from 2005 to 2014 is shown in Table 2. There were 44.81% of participants currently married, and the elderly who had been widowed for 21+ years accounted for 21.80% in 2005. There was a significant difference in cognitive scores across different marital status groups in each wave (*p* < 0.001). Those who had been widowed for 21+ years had the worst cognitive function in each wave.

Table 3 shows the results of multilevel growth models used to explore the association between widowhood status and cognitive function in all participants and different gender groups from 2005 to 2014 (the result of control variables is shown in Supplementary Table S2). In model 1, the widowed individuals were more likely to experience cognitive decline (B = − 0.440, 95% CI −0.727 to −0.152) after adjusting for the covariates. The results stratified by gender indicated that there was a gender difference in the relationship between widowhood and cognitive function. The widowhood status had a significant negative impact on cognitive function in men (B = −0.722, 95% CI −1.104 to −0.339). Widowed men had a total cognition score that was 0.722 lower than that of married men. However, the relationship is not significant in women, which suggested that the men were more likely be affected by the loss of a spouse than the women.

Table 4 shows the association between widowhood duration and cognitive function after adjusting for the covariates based on longitudinal data from 2005 to 2014 (the result of control variables is shown in Supplementary Table S3). A linear term of widowhood duration was included in model 2. The continuous variable of widowhood duration was negatively and significantly associated with the cognitive score in all participants (B = −0.028, 95% CI −0.038 to −0.019) and both women (B = −0.014, 95% CI −0.027 to −0.002) and men (B = −0.043, 95% CI −0.059 to −0.027) groups. The time since spousal loss was further categorized into six groups in model 3. The results suggested that being widowed for 21+ years (B = −1.109, 95% CI −1.488 to −0.730) had a significant negative effect on cognitive function, and widowers and widows had different trajectories of cognitive decline with increases in time since spouse loss. Compared with currently married men, men widowed for 5 years or less were more likely to have lower cognitive scores (B = −0.606, 95% CI −1.112 to −0.100), as were men widowed for 16–20 years (B = −0.937, 95% CI −1.685 to −0.190) and more than 21 years (B = −1.401, 95% CI −1.967 to −0.834). For women, only those who were widowed for more than 21 years (B = −0.655, 95% CI −1.186 to −0.124) had worse cognitive function than those currently married. The result showed that the elderly who were widowed for a long time were more likely to experience cognitive decline.

## 4. Discussion

In a society such as China, where people value parent–child relationships more than marriage relationships, the important role that spouses play in later life is often overlooked. Given the paucity of research about the association between widowhood and cognitive function among Chinese elderly individuals, especially the lack of research based on national representative samples with longitudinal data, the present study attempted to explore this association through a national population-based study with 9 years of follow-up data. Clear evidence was found that widowhood was a risk factor for cognitive decline among Chinese elderly individuals. Our conclusion was consistent with previous studies [26,27,29], which supported the protective effect of marriage on cognitive health. As mentioned above, the stress model and marital resource model might explain the mechanisms between widowhood and cognitive function [11,12,13,14].

The results of the gender stratification demonstrated that the association between widowhood and cognitive function was stronger in men than in women and was only statistically significant in men. This is consistent with the conclusions of a cross-sectional study of Chinese elderly individuals in Singapore [29]. Role theory can be used to explain the gender difference in the widowhood effect and its influence mechanism [39,40]. In traditional marriage, men are primarily financially responsible, while women often play the roles of caregiver and emotional supporter [41]. Chinese women tend to marry older men [42], thus, relatively young wives naturally become their husbands’ daily caregivers in old age. Men are more dependent on their spouses to handle their life, such as housework and daily care [43]. However, women tend to have closer friendships and social networks and receive more social support from their children [29,43]. Chinese elderly people attach great importance to the support from children in their later life. It has been proven that support from children can mitigate the negative effects of widowhood [15]. Moreover, men are often expected to be strong and to not readily express their feelings based on traditional Chinese stereotypes [44]. The image of a “strict father” leads to certain barriers for men to establish emotionally close relations with children [45]. Previous studies have confirmed that Chinese elderly women are more likely to benefit from close relationships with their children than men [46], which may protect their cognitive function after being widowed. However, our conclusion differs from studies in Mexico and India, which supported the view that widowhood would do more damage to elderly women rather than men [22,23]. The mixed results may be related to differences in family function and gender conception across countries.

One of the most important and surprising findings of this study was that widowhood duration indeed mattered in the relationship between widowhood and cognitive function. The longer widowhood duration might result in obviously decline of cognitive function, and the trajectories of cognitive decline was different across gender groups. In the early period of spouse loss, the men showed cognitive decline, while the women did not. Men were suggested to be more likely to face the dilemmas of being unable to handle housework, having a lack of caregivers and experiencing depressive symptoms [47], which might impose stress on their cognitive function. However, unlike some existing studies [48,49], we found no evidence that the negative effects of widowhood diminished over time. Instead, the association was found to be more pronounced in elderly individuals with longer widowhood durations in both men and women, which was consistent with a study with an average follow-up of 21 years [50]. This might be because brain changes occur slowly [30], and the long-term accumulation of a lack of cognitive stimulation results in an obvious decline in cognitive function. In addition, another possible reason was suggested that people who have been widowed for a long time were likely to receive less financial support from their children than those who have been widowed recently in China [51]. The scarcity of social support and economic resources might cause a decline in the cognitive function of long-term widowed elderly individuals. The indicator of widowhood duration requires special attention in future studies. Further research is needed to determine how the mechanisms linking widowhood to cognitive function vary over the course of widowhood so as to take targeted measures to reduce the secondary chain reaction of widowhood.

Evaluating the association between widowhood and cognitive function is useful in health policies aimed at the prevention of dementia. Numerous widowed elderly people in China are a vulnerable group, but they have not obtained enough attention yet. On the one hand, children should take more responsibility to take care of widowed elderly, especially the emotional comfort, to mitigate negative effects of widowhood on cognitive function. On the other hand, the government should provide resources and support to help family care for the widowed elderly so as to alleviate the negative effects of widowhood. Given the fact that widowed men are more likely to be negatively affected by spouse loss than women, both Chinese government and families should pay more attention to daily care and psychological intervention for widowed men. In addition, a strengthened social safety net should be built, especially for the widowed elderly without family care.

Nevertheless, the results should be interpreted cautiously for several limitations. First, previous studies have suggested that widowhood might increase the mortality risk of elderly individuals [52], which caused the problem of survivor bias in our present samples. That is, the widowed elderly individuals in our study may be survivors with relatively better health status, thus, resulting in an underestimation of the negative effect of widowhood on cognitive function. Second, there may be covariates that affect the cognitive function of elderly individuals that were not included in the present study, such as marriage quality and length of marriage. It has been proven that widowed elderly individuals with higher marital quality also had a higher level of depression than those who reported lower marital quality [53]. Third, we did not take psychological adjustment process into consideration to further explain the mechanism between widowhood duration and cognitive function, which might be explored in future studies.

## 5. Conclusions

This study shed light on the association between widowhood and cognitive function among Chinese elderly people. Especially, we provided clear evidence that gender and widowhood duration really mattered in this association, which contributes to the existing literature by improving understanding of the complexity of the linkage between widowhood and cognitive function. In conclusion, widowhood was significantly related to the decline in cognitive function among Chinese elderly individuals while adjusting for many demographic and socioeconomic characteristics. This association was statistically significant among men but not women. The widowhood duration also impacted the cognitive function. Recent and long-term widowhood predicted worse cognitive function in men, while only being widowed for more than 21 years was associated with cognitive decline in women. The results raise great concerns for the numerous elderly people in China, who might have a higher risk of cognitive decline. Psychosocial interventions, care services, and emotional comfort should be targeted to widowed elderly individuals to protect them from dementia, especially widowed men and those who have been widowed for a long time.

## Figures and Tables

**Table 1:** Descriptive statistics of the study sample (2005–2014).

Variables	Measurement	Number (%)/Mean (SD)			
		2005 (*n* = 5872)	2008 (*n* = 5872)	2011 (*n* = 3172)	2014 (*n* = 2034)
Cognitive function	Continuous measurement	25.12 (6.83)	22.63 (8.98)	23.65 (8.11)	24.25 (7.59)
Marital status	Widowed = 1	3267 (55.64)	3634 (61.89)	1814 (57.19)	1146 (56.34)
	Married = 0	2605 (44.36)	2238 (38.11)	1358 (42.81)	888 (43.66)
Age	Continuous measurement	80.64 (10.32)	83.79 (10.35)	82.87 (8.59)	83.33 (7.36)
Gender	Man = 1	2595(44.19)	2595(44.19)	1439 (45.37)	915 (44.99)
	Woman = 0	3277(55.81)	3277(55.81)	1733 (54.63)	1119 (55.01)
Education (years)	Continuous measurement	2.36 (3.65)	2.36 (3.65)	2.59 (3.75)	2.73 (3.75)
Place of residence	Rural = 1	3464 (58.99)	3297(56.15)	1350 (42.56)	803 (39.48)
	City = 0	2408 (41.01)	2575(43.85)	1822 (57.44)	1231 (60.52)
ADL	Any ADL limitations = 1	697 (11.87)	986 (16.79)	695 (21.91)	438 (21.53)
	No ADL limitations = 0	5175 (88.13)	4886 (83.21)	2477 (78.09)	1596 (78.47)
Chronic disease	Yes = 1	2377 (40.48)	2777 (47.29)	1646 (51.89)	1104 (54.28)
	No = 0	3495 (59.52)	3095 (52.71)	1526 (48.11)	930 (45.72)
Income sufficient for daily expense	Yes = 1	4546 (77.42)	4599 (78.32)	2442 (76.99)	1622 (79.74)
	No = 0	1326 (22.58)	1273 (21.68)	730 (23.01)	412 (20.26)
Children’s financial support	Yes = 1	5033 (85.71)	5029 (85.64)	2393 (75.44)	1418 (69.71)
	No = 0	839 (14.29)	843 (14.36)	779 (24.56)	616 (30.29)
Smoked past	Yes = 1	1991 (33.91)	1991 (33.91)	1109 (34.96)	699 (34.37)
	No = 0	3881 (66.09)	3881 (66.09)	2063(65.04)	1335 (65.63)
Drank past	Yes = 1	4176 (71.12)	4176 (71.12)	2270 (71.56)	1451 (71.34)
	No = 0	1696 (28.88)	1696 (28.88)	902 (28.44)	583 (28.66)
Exercised past	Yes = 1	2020 (34.40)	2020 (34.40)	1067 (33.64)	699 (32.89)
	No = 0	3852 (65.60)	3852 (65.60)	2105 (66.36)	1365 (67.11)
Living with others	Yes = 1	5038 (85.80)	4918 (83.75)	2675 (84.33)	1683 (82.74)
	No = 0	834 (14.20)	954 (16.25)	497 (15.67)	351 (17.26)

**Table 2:** Cognitive function based on marital status from 2005 to 2014.

**Marital Status**	**T = 2005**			**T = 2008**		
	**Number (%)**	**Cognitive Function** **Mean (SD)**	***p***	**Number (%)**	**Cognitive Function** **Mean (SD)**	***p***
Currently Married	2631 (44.81)	27.18 (4.70)	<0.001	2238 (38.11)	25.90 (6.57)	<0.001
Widowed 0 to 5 years	609 (10.37)	25.65 (5.92)		752 (12.81)	23.76 (7.60)	
Widowed 6 to 10 years	510 (8.69)	24.77 (6.35)		450 (7.66)	22.18 (9.04)	
Widowed 11 to 15 years	444 (7.56)	24.21 (7.54)		503 (8.57)	21.77 (8.95)	
Widowed 16 to 20 years	398 (6.78)	23.13 (7.90)		419 (7.14)	20.49 (9.79)	
Widowed 21+ years	1280 (21.80)	21.68 (8.65)		1510 (25.72)	18.25 (10.30)	
**Marital status**	**T = 2011**			**T = 2014**		
	**Number (%)**	**Cognitive function** **Mean (SD)**	***p***	**Number (%)**	**Cognitive function** **Mean (SD)**	***p***
Currently Married	1427 (44.99)	26.06 (5.99)	<0.001	903 (44.40)	26.20 (5.91)	<0.001
Widowed 0 to 5 years	278 (8.76)	24.12 (7.98)		153 (7.52)	25.44 (6.58)	
Widowed 6 to 10 years	273 (8.61)	23.21 (7.78)		244 (12.00)	23.61 (8.13)	
Widowed 11 to 15 years	254 (8.01)	22.89 (8.40)		159 (7.82)	23.80 (7.59)	
Widowed 16 to 20 years	247 (7.79)	21.89 (8.88)		164 (8.06)	22.77 (8.29)	
Widowed 21+ years	693 (21.85)	19.60 (9.71)		411 (20.21)	20.67 (9.03)	

**Table 3:** The association between widowhood status and cognitive function.

	Total (*n* = 5872)			Women (*n* = 3277)			Men (*n* = 2595)		
Parameter	B	SE	95% CI	B	SE	95% CI	B	SE	95% CI
Model 1									
Currently married (Ref.)									
Widowed	−0.440 **	0.147	[−0.727, −0.152]	−0.200	0.217	[−0.625, 0.225]	−0.722 ***	0.195	[−1.104, −0.339]
Intercept	23.479 ***	0.267	[22.956, 24.003]	22.537 ***	0.365	[21.822, 23.251]	25.650 ***	0.383	[24.899, 26.400]
Model Fit									
Log likelihood	−55,547			−31,285			−24,165		
AIC	111,128			62,603			48,363		
BIC	111,259			62,718			48,474		

** *p* < 0.01; *** *p* < 0.001; B, estimated coefficient, SE, standard error, CI, confidence interval; AIC, Akaike information criterion, BIC, Bayesian information criterion.

**Table 4:** The association between widowhood duration and cognitive function.

	Total (*n* = 5872)			Women (*n* = 3277)			Men (*n* = 2595)		
	B	SE	95% CI	B	SE	95% CI	B	SE	95% CI
Model 2									
Widowhood duration (Years)	−0.028 ***	0.005	[−0.038, −0.019]	−0.014 *	0.006	[−0.027, −0.002]	−0.043 ***	0.008	[−0.059, −0.027]
Intercept	23.573 ***	0.241	[23.100, 24.047]	22.614 ***	0.325	[21.977, 23.252]	25.530 ***	0.358	[24.828, 26.232]
Model Fit									
Log likelihood	−55,534			−31,283			−24,158		
AIC	111,102			62,599			48,348		
BIC	111,233			62,713			48,459		
Model 3									
Currently married (Ref.)									
Widowed 0 to 5 years	−0.111	0.188	[−0.479, 0.258]	0.199	0.273	[−0.337, 0.735]	−0.606 *	0.258	[−1.112, −0.100]
Widowed 6 to 10 years	−0.326	0.208	[−0.733, 0.081]	−0.226	0.287	[−0.789, 0.337]	−0.428	0.309	[−1.033, 0.177]
Widowed 11 to 15 years	−0.383	0.220	[−0.813, 0.048]	−0.293	0.297	[−0.876, 0.290]	−0.359	0.341	[−1.028, 0.311]
Widowed 16 to 20 years	−0.431	0.233	[−0.888, 0.025]	−0.044	0.309	[−0.650, 0.562]	−0.937 *	0.381	[−1.685, −0.190]
Widowed 21+ years	−1.109 ***	0.193	[−1.488, −0.730]	−0.655 *	0.271	[−1.186, −0.124]	−1.401 ***	0.289	[−1.967, −0.834]
Intercept	23.617 ***	0.267	[23.094, 24.140]	22.632 ***	0.364	[21.918, 23.346]	25.654 ***	0.380	[24.909, 26.400]
Model Fit									
Log likelihood	−55,533			−31,280			−24,159		
AIC	111,108			62,601			48,359		
BIC	111,271			62,744			48,497		

* *p* < 0.05; *** *p* < 0.001; B, estimated coefficient, SE, standard error, CI, confidence interval; AIC, Akaike information criterion, BIC, Bayesian information criterion.

## Data Availability

The data that support the findings of this study are openly available in the Peking University Open Research Data at https://opendata.pku.edu.cn/dataverse/CHADS.

## Supplementary Materials

The following are available online at https://www.mdpi.com/article/10.3390/healthcare9080991/s1, Table S1: The Chinese version of mini-mental state examination adopted in CLHLS. Table S2. The association between widowhood status and cognitive function. Table S3. The association between widowhood duration and cognitive function.
