# Supplementary material for: The Association between Widowhood and Cognitive Function among Chinese Elderly People: Do Gender and Widowhood Duration Make a Difference?
Source: Healthcare (Basel). 2021 Aug 4;9(8):991. doi: 10.3390/healthcare9080991 (PMC8392527; doi:10.3390/healthcare9080991)
Supplement: Supplementary file 1 [file healthcare-09-00991-s001.zip › healthcare-1296570-supplementary.pdf]

Supplementary Tables

Table S1. The Chinese version of mini-mental state examination adopted in CLHLS.

Domain	Question	Score (Total = 30)
Orientation	What time of day is it right now (morning, afternoon, evening)?	1
	What is the animal year of this year?	1
	What is the date (day and month) of the mid-autumn festival?	1
	What is the season right now?	1
	What is the name of this county or district?	1
Naming foods	Please name as many kinds of food as possible in 1 minute (1 point for each food and 7 points for those who name 7 or more foods).	7
Registration	Table, apple, cloth. Please repeat these 3 objects.	3
Attention and calculation	I will ask you to spend $3 from $20, then you must spend $3 from the number you arrived at and continue to spend $3 until you are asked to stop.	5
Copy a figure	The individual is asked to draw a figure of overlapping pentagons.	1
Recall	Name the 3 objects learned earlier (table, apple, and cloth).	3
Language	Naming pen and watch.	2
	Repeating the following sentence: "What you plant is what you will get."	1
	The individual is asked to follow the interviewer's instruction: "Take the paper using your right hand, fold it in the middle using both hands, and place the paper on the floor."	3

Table S2. The association between widowhood status and cognitive function.

	Total (n = 5872)	Women (n = 3277)	Men (n = 2595)
Fixed Effect			
Widowed	-0.440 **	-0.200	0.722 ***
Age	-0.281 ***	-0.316	-0.222 ***
Gender	1.144 ***	-	-
Education (years)	0.193 ***	0.286 ***	0.160 ***
Place of residence	-0.514 ***	-0.310 *	-0.694 ***
ADL	-6.084 ***	-5.960 ***	-6.259 ***
Chronic disease	0.196	0.359	-0.062
Income sufficient for daily expense	1.244 ***	1.394 ***	1.021 ***
Children's financial support	0.344 **	0.667 ***	-0.027
Smoked past	0.028	0.271	-0.017
Drank past	-0.371 *	-0.570 *	-0.193
Exercised past	0.381 **	0.454 *	0.167
Living with others	-0.486 **	-0.420 *	-0.465
Intercept	23.479 ***	22.537 ***	25.650 ***
Random Effect			
Linear slope SD	0.220	0.219	-24165
Intercept SD	2.503	2.767	48363

Residual SD	5.714	5.969	48474
Model Fit			
Log likelihood	-55547	-31285	-24165
AIC	111128	62603	48363
BIC	111259	62718	48474
Number of observations	16950	9406	7544
Number of individuals	5872	3277	2595

*$p < 0.05$; **$p < 0.01$; ***$p < 0.001$; AIC Akaike information criterion, BIC Bayesian information criterion.

Table S3. The association between widowhood duration and cognitive function.

	**Total (n = 5872)**	**Women (n = 3277)**	**men (n = 2595)**
Fixed Effect			
Currently married (Ref.)			
Widowed 0 to 5 years	-0.111	0.199	-0.606 *
Widowed 6 to 10 years	-0.326	-0.226	-0.428
Widowed 11 to 15 years	-0.383	-0.293	-0.359
Widowed 16 to 20 years	-0.431	-0.044	-0.937 *
Widowed 21+ years	-1.109 ***	-0.655 *	-1.401 ***
Age	-0.269 ***	-0.305 ***	-0.219 ***
Gender	1.022 ***	-	-
Education (years)	0.192 ***	0.286 ***	0.157 ***
Place of residence	-0.512 ***	-0.316 *	-0.678 ***
ADL	-6.072 ***	-5.960 ***	-6.240 ***
Chronic disease	0.176	0.340 *	-0.079
Income sufficient for daily expense	1.239 ***	1.389 ***	1.016 ***
Children's financial support	0.331 *	0.659 ***	-0.033
Smoked past	0.034	0.266	-0.011
Drank past	-0.358 *	-0.543 *	-0.200
Exercised past	0.370 **	0.448 *	0.168
Living with others	-0.450 **	-0.400 *	-0.425
Intercept	23.617 ***	22.632 ***	25.654 ***
Random Effect			
Linear slope SD	0.219	0.218	0.206
Intercept SD	2.506	2.770	2.145
Residual SD	5.711	5.965	5.373
Model Fit			
Log likelihood	-55533	-31280	-24159
AIC	111108	62601	48359
BIC	111271	62744	48497
Number of observations	16950	9406	7544
Number of individuals	5872	3277	2595

*$p < 0.05$; **$p < 0.01$; ***$p < 0.001$; AIC Akaike information criterion, BIC Bayesian information criterion.
